# Ejection Fraction-Related Differences in Left Ventricular and Atrial Strain Indices Among Pediatric Fontan Circulation with Systemic Left Ventricle Morphology

**DOI:** 10.3390/diagnostics16010171

**Published:** 2026-01-05

**Authors:** Carmen Corina Șuteu, Amalia Fagarasan, Nicola Suteu, Andreea Cerghit-Paler, Liliana Gozar, Cristina Oana Mărginean, Maria Oana Săsăran, Mihaela Iancu

**Affiliations:** 1 Department of Pediatrics III, Faculty of Medicine, “George Emil Palade” University of Medicine, Pharmacy, Science, and Technology of Târgu Mureș, 540136 Târgu Mureș, Romanialili_gozar@yahoo.com (L.G.); 2 Department of Pediatric Cardiology, Emergency Institute for Cardiovascular Diseases and Transplantation, 540136 Târgu Mureş, Romania; 3 Department of Pediatrics I, “George Emil Palade” University of Medicine, Pharmacy, Science, and Technology of Târgu Mureș, 540136 Târgu Mureș, Romania; 4 Department of Pediatrics III, Faculty of Medicine English, “George Emil Palade” University of Medicine, Pharmacy, Science, and Technology of Târgu Mureș, 540136 Târgu Mureș, Romania; 5 Department of Medical Informatics and Biostatistics, Iuliu Hațieganu University of Medicine and Pharmacy, 400012 Cluj-Napoca, Romania

**Keywords:** pediatric Fontan circulation, systemic left ventricle, ejection fraction, strain echocardiography

## Abstract

**Background**: Ventricular function assessments in Fontan patients remain challenging. Ejection fraction (EF) lacks sensitivity for early dysfunction, and the roles of strain and advanced imaging in systemic left ventricle (LV) physiology are not fully defined. We aimed to compare (i) LV and atrial strain indices between pediatric Fontan patients with preserved EF (P-LVEF) versus reduced EF (R-LVEF) and (ii) echocardiographic global longitudinal strain, segmental longitudinal strain indices, and conventional 2D and 3D echocardiographic parameters through cardiac morphology. **Methods**: Pediatric patients with Fontan circulation and systemic LV morphology underwent clinical, hemodynamic, and multimodality echocardiographic evaluation, including 2D/3D parameters, global and segmental LV strain, and left atrial strain. Outcomes were analyzed according to EF status and congenital morphology. Significant results from multiple comparisons were followed by post hoc analysis, where appropriate. **Results**: Patients with a reduced EF exhibited a worse clinical status, a higher pulmonary vascular resistance index, and greater systemic congestion compared with those with a preserved EF. Conventional 2D indices showed no significant differences between the two studied groups except for LV end-systolic volume (ESV) (*p* = 0.0315) and LV end-systolic longitudinal diameter (ESL) (*p* = 0.0024), which showed higher values in the R-LVEF group. Although the relative frequency of impaired deformation was higher in Fontan patients with an unbalanced atrioventricular canal compared with the Fontan patients with a tricuspid atresia + pulmonary stenosis + ventricular septal defect, the difference did not reach statistical significance (*p* = 0.1365). Most segmental longitudinal strain values were not significantly different across patients with different cardiac morphology, except for the basal anterior segment and apical inferoseptal segment (*p* < 0.05). **Conclusions**: In pediatric Fontan patients with systemic LV morphology, a reduced EF was associated with a worse clinical and hemodynamic status. Conventional echocardiographic indices showed a limited ability to differentiate between the compared groups. Although no statistically significant differences were detected between pediatric Fontan patients with preserved EF and reduced EF, LV and atrial strain indices provided complementary information on ventricular–atrial interactions and myocardial deformation. These findings are exploratory and warrant confirmation in larger, prospective studies.

## 1. Introduction

Children with single-ventricle (SV) physiology who undergo Fontan palliation remain at a high risk for long-term morbidity and mortality. The Fontan circulation, which directs systemic venous return directly to the pulmonary arteries without a subpulmonary ventricle, is characterized by a passive pulmonary blood flow, chronically elevated venous pressures, and reduced ventricular preload. Ventricular dysfunction, arising from adverse loading conditions and intrinsic structural abnormalities, is a principal driver of Fontan failure, leading to impaired systolic–diastolic mechanics, reduced functional reserve, and maladaptive remodeling [1,2,3]. However, the accurate assessment of ventricular performance in this population is complicated by heterogeneous morphology and complex three-dimensional geometry.

Conventional two-dimensional echocardiography (2D) is widely used for surveillance but is limited by load dependence, geometric assumptions, and a reduced sensitivity to subtle functional differences [1,4]. Three-dimensional (3D) echocardiography allows for volumetric quantification without geometric assumptions and has demonstrated a closer agreement with cardiac magnetic resonance imaging (CMR) than 2D measures [5,6,7,8]. Importantly, both 2D and 3D volumetric indices primarily reflect global pump performance and may fail to detect early myocardial impairment.

Speckle-tracking echocardiography (STE) enables the angle-independent quantification of myocardial deformation and has been reported to detect functional abnormalities not captured by conventional indices. In Fontan patients, global longitudinal strain (GLS) and strain rate have been reported to correlate with invasive hemodynamic and CMR-derived parameters and may provide information complementary to ejection fraction (EF) [9,10]. Morphology-specific vulnerability is evident, with the systemic right ventricle (RV) demonstrating earlier and more pronounced strain abnormalities compared with the systemic left ventricle (LV) [11,12]. In addition, atrial strain analysis has emerged as clinically relevant: reductions in reservoir and conduit strain are associated with impaired exercise capacity, arrhythmias, transplantation, and mortality, and are detectable even in clinically stable pediatric patients [13,14,15,16].

Although STE is increasingly applied in Fontan patients, data stratified by EF remain scarce. Evidence from pediatric and adult cohorts shows that strain indices, particularly longitudinal strain and strain rate, are impaired despite the preserved EF, revealing subclinical systolic dysfunction [17,18,19,20]. In adults, impaired GLS, typically <−18%, predicts progressive ventricular deterioration and adverse events, underscoring that EF alone underestimates myocardial disease burden [20]. In pediatric populations, the relationship between ventricular and atrial strain indices and EF remains insufficiently characterized. The limited available evidence does not clarify whether strain abnormalities are present even in those with a preserved EF or are restricted to reduced EF states [17]. This gap is clinically relevant, as EF-based stratification continues to inform surveillance strategies in clinical practice.

Objective. We aimed to compare (i) LV and atrial strain indices between pediatric Fontan patients with preserved EF (P-LVEF) versus reduced EF (R-LVEF) and (ii) echocardiographic global longitudinal strain, segmental longitudinal strain indices, and conventional 2D and 3D echocardiographic parameters through cardiac morphology.

## 2. Materials and Methods

### 2.1. Study Design and Population

This current observational analytical study was based on an established single-center Fontan cohort previously described in detail [21]. The original cohort consisted of 22 pediatric patients with Fontan circulation and systemic LV morphology followed at the Pediatric Cardiology Department of the Emergency Institute for Cardiovascular Diseases and Transplantation, Târgu-Mureș, Romania.

For the present investigation, we included all patients from the previously published cohort and performed a secondary analysis of the previously acquired echocardiographic datasets.

The local ethics committee approved the secondary analysis of stored anonymized data, and informed consent had been obtained from the legal guardians in accordance with the Declaration of Helsinki at the time of original data collection.

### 2.2. Clinical Data Collection

Clinical parameters were retrieved from medical records and included demographics, anthropometric measurements, and New York Heart Association functional class (NYHA FC). Surgical history, anatomical details, and paraclinical findings relevant to Fontan physiology were also reviewed. Pulmonary artery anatomy was assessed using established morphometric indices derived from pre-Fontan cardiac catheterization. The Nakata index was calculated as the sum of the cross-sectional areas of the right and left pulmonary arteries indexed to body surface area (mm^2^/m^2^), while the McGoon index was defined as the sum of the diameters of the right and left pulmonary arteries divided by the diameter of the descending aorta [22,23]. These indices were used to characterize pulmonary artery development and were included in the comparative analysis.

### 2.3. Echocardiographic Acquisition and Data Processing

All echocardiographic examinations had been performed using a Philips EPIQ CVx platform (Philips, Andover, MA, USA) equipped with an X5-1 transducer. The original imaging protocol adhered to the American Society of Echocardiography (ASE) pediatric recommendations [24]. For the current study, all analyses were performed offline by re-evaluating the stored datasets using Philips QLab 15.0 software (material number 453562090801).

Two-dimensional Echocardiography. Conventional measurements included mitral annular plane systolic excursion (MAPSE), Doppler-derived transmitral inflow velocities (E_mi and A_mi), their ratio (E_mi/A_mi), tissue Doppler mitral annular velocities (E′_mi, A′_mi, S_mi), the ratio of early mitral inflow to early annular velocity (E_mi/E′_mi), and LV isovolumic contraction and relaxation times (IVCT, IVRT). All Doppler and tissue Doppler measurements were averaged over three cardiac cycles.

Three-dimensional Echocardiography. Full-volume 3D datasets obtained from multi-beat ECG-gated acquisitions were used to derive LV end-diastolic volume (EDV), end-systolic volume (ESV), ejection fraction (EF), stroke volume (SV), LV mass, and longitudinal LV diameters (EDL, ESL). Datasets exhibiting stitching artifacts, poor endocardial tracking, or excessive heart rate variability were excluded.

Speckle-Tracking Echocardiography. Speckle-tracking analyses were performed from previously stored apical views with adequate frame rates (typically 50–90 frames/s). LV GLS was calculated as the mean peak negative longitudinal strain from all available LV segments. Left atrial (LA) strain was assessed in the apical four-chamber view using an R-wave-based zero reference. Reservoir, conduit, and contractile strain components were derived from the corresponding strain curves. Only images with complete endocardial border visualization and acceptable tracking quality were selected.

### 2.4. Study-Specific Stratification

For the purpose of this secondary analysis of data, patients were stratified according to LV systolic function, assessed using 3D echocardiography: preserved EF (P-LVEF group): ≥50% and reduced EF (R-LVEF group): <50%. Echocardiographic parameters, including strain indices, volumetric measurements, and Doppler data, were compared between the two groups. Associations between 3D EF and deformation parameters were further explored.

### 2.5. Statistical Analysis

The distributions of continuous variables (clinical, conventional echocardiographic and speckle-tracking echocardiographic characteristics) were checked using univariate descriptive statistics, quantile–quantile plots, and Shapiro–Wilk’s test. All normally distributed variables were summarized as an arithmetic mean and standard deviation (SD), while variables that deviated from a Gaussian distribution were presented using a median with interquartile range (IQR): 25th percentile to 75th percentile. Qualitative variables were described using absolute and relative frequencies (%). Student’s *t*-test for independent samples or Wilcoxon rank sum test with continuity correction was used to compare demographic, anthropometric, and surgical characteristics between Fontan patients with R-LVEF and Fontan patients with P-LVEF groups. One-way ANOVA analysis, Welch F-test, or Kruskal–Wallis tests were used to compare distributions of echocardiographic global longitudinal strain and segmental longitudinal strain values through cardiac morphology. When a significant result was obtained from one-way ANOVA or Welch’s F test, post hoc pairwise comparisons were performed using Tukey’s HSD or Games–Howell tests, as appropriate. Frequency distributions of clinical symptoms, comorbidities, FC, and surgical procedures were compared between study groups using Fisher’s exact test. All descriptive and inferential statistical analyses were performed using R software version 4.5.1 (R Foundation for Statistical Computing, Vienna, Austria). All two-sided statistical tests used a significance level (α) set at 0.05. A significant result was achieved when the estimated significance level *p*-value < 0.05.

## 3. Results

### 3.1. Fontan Patients’ Characteristics

Twenty-two eligible patients after an extracardiac single LV Fontan procedure were included in the current study and were separated into two groups: those with a preserved LVEF (3D) (P-LVEF group, *n* = 15) and those with a reduced LVEF (3D) (R-LVEF group, *n* = 7). We found no significant differences in age and body surface area distributions between the two groups, as shown in Table 1. Comorbidity profiles were comparable across groups (*p* = 0.5227), the most frequent comorbidities being iron deficiency anemia, observed in 66.7% in the P-LVEF group vs. 42.9% in the R-LVEF group, thrombophilia (33.3% vs. 71.4%), polycythaemia (40% vs. 57.1%), pulmonary hypertension associated with univentricular heart physiopathology (PH) (13.4% vs. 71.4%), and thrombocytopenia (13.3% vs. 57.1%). Thirteen subjects (86.7%) in the P-LVEF group were classified as NYHA FC II, while two patients (13.3%) in the P-LVEF group were in NYHA FC III. In the R-LVEF group, all seven patients (100%) were in NYHA FC III.

Among the Fontan patients, the most common clinical symptoms were cyanosis (11, 50%), hepatomegaly (7, 31.82%), and edema (3, 13.64%). Significant differences in the frequency of edema (*p* = 0.0227) and hepatomegaly (*p* = 0.0136) were observed between Fontan patients with P-LVEF and those with R-LVEF (Table 2). The pulmonary vascular resistance index (PVRi) was also significantly different between groups (*p* = 0.0348), reflecting differences in pulmonary vascular resistance, whereas the Nakata index showed a similar distribution between groups (*p* = 0.1265).

### 3.2. Comparison of Conventional Left Ventricle 2D and 3D Echocardiographic Characteristics Between Fontan Patients with R-LVEF and Those with P-LVEF

We found no significant differences in standard 2D and 3D echocardiographic parameters between the two studied groups except for LV ESV (*p* = 0.0315) and LV ESL (*p* = 0.0024), which showed higher values in the R-LVEF group (Table 3).

### 3.3. Strain Analysis of the Left Ventricle and Left Atrium: Fontan Patients with P-LVEF Versus Fontan Patients with R-LVEF

There were no significant differences in GLS and segmental longitudinal strain indices for the LV (Table 4). The left atrial reservoir strain did not reach statistical significance, but a trend was observed (*p* = 0.0941), with higher values noted in Fontan patients with R-LVEF.

### 3.4. Strain Analysis of the Left Ventricle and Left Atrium by Types of Congenital Malformations with SV Physiology

Most segmental longitudinal strain values were not significantly different among the three diagnostic groups except for the basal anterior segment and apical inferoseptal segment (Table 5). Pairwise post hoc comparisons showed that Fontan patients with tricuspid TA+PS+VSD had significantly lower mean longitudinal strain values for the basal anterior segment than Fontan patients with an unbalanced AVC (adjusted *p* = 0.008, mean, 95% CI: −18.7 [−25.2, −12.1] vs. −5.83 [−10.1, −1.59]), indicating a better myocardial deformation. The post hoc analysis also identified a tendency to statistical significance for the difference in mean longitudinal strain values of the apical inferoseptal segment between Fontan patients with DILV and those with TA+PS+VSD (adjusted *p* = 0.058), with a better systolic function observed in the TA+PS+VSD group (Table 5).

### 3.5. Conventional Left Ventricle 2D and 3D Echocardiographic Characteristics by Types of Congenital Malformations with SV Physiology

Welch’s F-test indicated a significant group effect in peak mitral inflow velocity during early diastole (E_mi wave) (*p* = 0.022), mitral annular systolic velocity (S_mi wave), and LVSV (Table 6). Games–Howell post hoc comparisons showed that Fontan patients with DILV differed significantly from Fontan patients with TA+PS+VSD in terms of E_mi velocity (adjusted *p* = 0.013), while the other two diagnostic group showed no significant difference (adjusted *p* = 0.577). Regarding S_mi and SV, the post hoc analysis revealed a significant difference exclusively for the contrast between Fontan patients with TA+PS+VSD and Fontan patients with unbalanced AVC (adjusted *p* = 0.031 and adjusted *p* = 0.014, respectively).

## 4. Discussion

In this study, we evaluated ventricular and atrial function in a homogeneous cohort of extracardiac Fontan patients with a single LV morphology (DILV, TA+PS+VSD, unbalanced AVC). A stratification by LV systolic function assessed using 3D echocardiography was associated with distinct clinical and hemodynamic profiles, highlighting an association between ventricular performance, pulmonary vascular physiology, and systemic clinical features. Our results demonstrated that patients with R-LVEF were uniformly symptomatic (FC III), whereas most patients with P-LVEF were in FC II. The R-LVEF group demonstrated significantly higher PVRi, more frequent systemic congestion (edema, hepatomegaly), and elevated hematocrit values, consistent with the pathophysiological coupling of ventricular dysfunction and PVR in the Fontan circulation [25,26,27]. Even modest systolic impairment may be associated with increased venous hypertension, limited preload, and impaired systemic perfusion in the Fontan circulation. Although pulmonary artery dimensions (Nakata, McGoon indices) were comparable between groups, PVRi was significantly different between study groups, with elevated values in R-LVEF patients. This finding suggests that endothelial and microvascular factors, beyond anatomical pulmonary artery dimensions, may contribute to Fontan hemodynamics [28]. The higher relative frequency of pulmonary hypertension, thromboembolic events, and atrioventricular valve regurgitation in the R-LVEF group further illustrates the multifactorial burden of Fontan failure. Thromboembolic complications may not only result from low-flow states but also perpetuate pulmonary vascular remodeling, worsening ventricular loading conditions [29]. The association of hepatomegaly and elevated hematocrit with R-LVEF reflects the progression of Fontan-associated liver disease and chronic cyanosis, both increasingly recognized as key determinants of late morbidity and mortality [30,31,32]. Our findings highlight the precarious balance between ventricular contractility, pulmonary vascular resistance, and systemic adaptation in the Fontan circulation. Even subtle reductions in systolic function markedly increase the risk of systemic congestion, hepatic dysfunction, and thromboembolic complications. Early recognition and targeted preventive strategies are therefore essential to preserve circulatory efficiency and improve long-term survival.

In the current cohort of pediatric Fontan patients with a single LV morphology, conventional 2D echocardiographic parameters showed minimal group differences, with only ESV and ESL significantly different between study groups, with higher values observed in patients with R-LVEF. This finding is consistent with the limited ability of standard 2D indices to differentiate systolic function across EF-based groups in Fontan physiology. In contrast, 3D echocardiography provides a more reliable volumetric assessment and closer agreement with cardiac MRI [5,6]. The observation that only ESV and ESL differed between groups highlights the potential role of advanced imaging in further characterizing ventricular mechanics beyond conventional parameters. Real-time 3D echocardiography and deformation imaging may provide additional information on ventricular geometry and mechanics when conventional indices appear preserved [7]. Myocardial and atrial strain parameters may reflect aspects of ventricular–atrial interactions and preload dynamics relevant to Fontan physiology. Their integration with 3D volumetric analysis may improve risk stratification and guide timely interventions.

In this study, global and segmental LV longitudinal strain indices did not differ significantly between Fontan patients with P-LVEF and R-LVEF. This result is consistent with prior evidence showing that GLS does not consistently discriminate systolic function across stages of Fontan circulation [33]. Although GLS is sensitive to early myocardial dysfunction, its value as a surrogate for ventricular performance appears limited in patients with a dominant LV morphology, which is generally more resilient than right-sided morphologies [11]. The absence of statistically significant differences may reflect the limited sample size and the cross-sectional nature of the analysis. Despite these limitations, previous studies support the diagnostic and prognostic role of strain imaging in Fontan patients [9,10,11,21,34]. Strong correlations have been demonstrated between STE-derived strain and MRI-derived indices, and GLS has shown closer associations with invasive hemodynamics than EF [10,11]. Left atrial reservoir strain showed a non-significant trend toward higher values in patients with R-EF, possibly reflecting compensatory adaptation to elevated systemic venous pressures. A growing body of evidence highlights the central role of atrial mechanics in Fontan physiology, with associations described across surgical stages, Fontan connection types, exercise capacity, and clinical outcomes [13,14,15,16,34]. These observations support the concept that atrial strain may provide complementary functional information related to ventricular filling and venous congestion. Combining ventricular and atrial strain within a multimodality imaging approach has the potential to improve clinical monitoring and risk stratification, with relevance in single LV morphology.

When myocardial deformation was analyzed according to cardiac morphology, most segmental and global strain parameters did not differ significantly among diagnostic groups. However, patients with TA+PS+VSD exhibited significantly better deformation in the basal anterior segment compared with those with unbalanced AVC and a trend toward superior function in the apical inferoseptal segment compared with DILV. These findings suggest that, even within single LV morphologies, different anatomic subgroups may demonstrate heterogeneous regional deformation patterns. Previous studies have consistently reported that ventricular morphology influences outcomes after Fontan completion. Steflik et al. (2017) showed that ventricular morphology affects deformation indices and correlates with hemodynamic status, while Wilkinson et al. (2022) demonstrated a reduced deformation capacity in systemic right compared with systemic LV [11,12]. By focusing exclusively on patients with dominant LV morphology, the present study minimizes the confounding influence of mixed ventricular anatomy while permitting the evaluation of lesion-specific mechanical differences. Within this context, subgroup-related factors, particularly atrioventricular valve involvement in patients with unbalanced AVC, may influence myocardial mechanics. Notably, none of the patients with unbalanced AVC underwent additional atrioventricular valve repair or replacement procedures specifically targeting valve competence between Fontan completion and the echocardiographic assessment included in this study. Nevertheless, atrioventricular valve dysfunction has been associated with volume overload and altered ventricular mechanics in Fontan patients [4]. In contrast, patients with TA+PS+VSD demonstrated relatively preserved or even superior segmental strain, suggesting a more favorable myocardial adaptation. A similar variability across lesion types was reported by Shiraga et al. (2021), who noted that the imposition of Fontan physiology exerts heterogeneous effects on strain depending on underlying anatomy [35]. This supports the need for individualized follow-up strategies that account for both global ventricular function and lesion-specific mechanical adaptations. Although GLS did not differ among groups, the segmental variations we observed highlight the importance of detailed regional analysis. As previously demonstrated, strain abnormalities may precede overt declines in EF or clinical deterioration [9,35]. Thus, morphology-specific strain assessment may contribute to a more detailed functional characterization and support individualized surveillance strategies.

This study demonstrates morphology-specific differences in conventional echocardiographic parameters among Fontan patients with systemic LV physiology. Significant differences were observed in E_mi wave, S′_mi wave, and LV SV. Specifically, DILV patients exhibited higher E_mi velocities compared with TA+PS+VSD, whereas TA+PS+VSD patients demonstrated higher S′_mi and SV compared with unbalanced AVC. These findings highlight the influence of underlying anatomy on both systolic and diastolic performance. Prior studies have shown that ventricular morphology is a major determinant of myocardial mechanics and outcomes after Fontan completion [11,36]. The impaired systolic velocities and reduced SV in unbalanced AVC patients are clinically relevant, likely reflecting the adverse impact of atrioventricular valve regurgitation and altered geometry, consistent with Davis et al. (2019), who identified diastolic and valve function as key determinants of early Fontan outcomes [4]. In contrast, relatively preserved systolic indices in TA+PS+VSD suggest more favorable ventricular–valvular coupling, paralleling the superior segmental strain observed in this subgroup. The higher E_mi velocities seen in DILV patients may reflect abnormal diastolic loading or elevated filling pressures. Diastolic dysfunction is a recognized hallmark of Fontan physiology, and transmitral Doppler indices such as the E wave have been associated with systemic venous congestion, impaired exercise tolerance, and clinical deterioration [4,33,37,38]. Thus, these findings reinforce the prognostic importance of incorporating conventional Doppler and tissue Doppler measures into routine surveillance. Taken together, our results confirm that congenital morphology exerts a measurable influence on both systolic and diastolic echocardiographic parameters in Fontan patients. While GLS and volumetric indices provide global insights, conventional indices such as E velocity, S′, and SV appear sensitive to subgroup-specific adaptations and may enhance risk stratification. The morphology-specific integration of conventional and deformation imaging may therefore improve clinical assessments and guide individualized follow-up strategies.

### 4.1. Strengths, Novel Contributions, and Clinical Implications

This study examined a homogeneous cohort of extracardiac pediatric Fontan patients with systemic LV morphology, thereby minimizing confounding from mixed anatomies. By integrating clinical status, hemodynamics, and multimodal echocardiographic indices, including 2D/3D volumetrics and atrial/ventricular strain, it provides a comprehensive characterization of Fontan physiology. Our findings emphasize morphology-specific adaptations and highlight the complementary role of atrial mechanics. Importantly, the identification of segmental strain and conventional Doppler parameters as potential early markers of dysfunction underscores the value of multimodal surveillance. While LVEF remains clinically relevant, conventional 2D indices alone are insufficient for detecting early deterioration. Advanced modalities such as 3D echocardiography and STE may enhance diagnostic characterization by providing complementary structural and functional information, while atrial strain may offer additional insight into ventricular–atrial interactions and preload adaptations in Fontan physiology. Taken together, these observations support individualized follow-up strategies that account for both global function and congenital substrate, with the potential to improve risk stratification and guide timely interventions.

### 4.2. Limitations

This study has several limitations. The relatively small sample size may limit statistical power and may increase the likelihood of failing to detect true differences, particularly for strain-based analyses. Cardiac magnetic resonance imaging was not systematically available for the external validation of echocardiographic measurements, and the retrospective strain analysis may have been influenced by image quality. Subgroup analyses, including those involving atrioventricular valve pathology, should be interpreted cautiously due to the limited subgroup sample size and the possibility that unmeasured confounders may still affect the observed bivariate associations. Finally, the single-center design may limit generalizability, underscoring the need for larger, prospective multi-center studies.

## 5. Conclusions

In this cohort of pediatric Fontan patients with systemic LV morphology, a reduced EF was associated with a worse clinical status, higher PVRi, and more frequent signs of systemic congestion compared with a preserved EF. Conventional 2D echocardiographic indices demonstrated a limited ability to differentiate between EF-based subgroups, with significant differences observed only for selected volumetric parameters. Advanced echocardiographic techniques, including 3D volumetric assessment and atrial and ventricular strain analysis, did not reveal statistically significant differences between the groups; however, they provided complementary structural and functional information regarding ventricular–atrial interactions, regional myocardial deformation, and circulatory adaptation. Morphology-specific differences in the selected deformation and Doppler parameters suggest that the underlying congenital substrate and atrioventricular valve involvement may influence myocardial mechanics even within a dominant LV Fontan population. Due to the limited sample size, these findings should be regarded as exploratory. Larger, prospective, multi-center studies are required to assess the incremental clinical and prognostic value of strain-based and 3D echocardiographic indices in this population.

## Figures and Tables

**Table 1:** Demographic, anthropometric, and surgical characteristics of the studied groups.

Variable	Fontan Patients with P-LVEF (*n* = 15)	Fontan Patients with R-LVEF (*n* = 7)	*p*-Value
Age at echocardiogram (years), mean (SD)	10.00 (2.80)	11.86 (4.38)	0.2404
Age at Fontan procedure (years), median (IQR)	4 (4 to 5)	4 (3.9 to 5)	0.5841
Time interval between the Fontan intervention and echocardiography (years)	4 (2.1 to 9)	7 (5.5 to 11)	0.2157
BMI (kg/m^2^), median (IQR)	14.12 (13.63 to 15.47)	19.84 (16.34 to 20.36)	0.0319 *
BMI z-scores, mean (SD)	−0.81 (1.31)	−0.82 (0.54)	0.9734
BSA (m^2^), mean (SD)	1.11 (0.38)	1.20 (0.28)	0.5840
BSA z-scores, mean (SD)	−0.81 (1.31)	−0.82 (0.54)	0.9734
SBP (mmHg), mean (SD)	107 (9.10)	110 (8.95)	0.9614
DBP (mmHg), mean (SD)	64 (7.82)	69 (7.37)	0.1938
Male sex, *n* (%)	9 (60)	5 (71.4)	1.000
Diagnosis, *n* (%)			0.1365
DILV	6 (40)	1 (14.3)	
TA+PS+VSD	7 (46.7)	2 (28.6)	
Unbalanced AVC	2 (13.3)	4 (57.1)	
Functional class, *n* (%)			0.0002 *
II	13 (86.7)	0 (0)	
III	2 (13.3)	7 (100)	
Surgical procedures			
Open fenestration, *n* (%)	8 (53.3)	5 (71.4)	0.6478
LPA dilation/stent, *n* (%)	5 (33.3)	3 (42.9)	1.000
RPA dilation/stent, *n* (%)	4 (26.7)	2 (28.6)	1.000
Closure of fenestration, *n* (%)	1 (6.7)	1 (14.3)	1.000
Closure of veno-venous collaterals, *n* (%)	2 (13.3)	2 (28.6)	0.5646
Closure of arterio-venous collaterals, *n* (%)	4 (26.7)	3 (42.9)	0.6302
CoAo surgery, *n* (%)	2 (13.3)	1 (14.3)	1.000
Comorbidities, *n* (%)	12 (80)	7 (100)	0.5227
AV valve regurgitation			0.0536
Mild	13 (86.7)	3 (42.9)	
Moderate	2 (13.3)	4 (57.1)	
Prolonged hospitalization after stage I palliation, *n* (%)	0 (0)	3 (42.9)	0.0227 *
Unbalanced AVC, *n* (%)	2 (13.3)	4 (57.1)	0.0536
Heterotaxy syndrome, *n* (%)	2 (13.3)	1 (14.3)	1.000
Hypoxemia after PCPC, *n* (%)	9 (60)	7 (100)	0.1206
High RA pressure, *n* (%)	1 (6.7)	5 (71.4)	0.0043 *
Elevated preoperative PAP (>15 mmHg), *n* (%)	1 (6.7)	2 (28.6)	0.2273
Presence of fenestration TCPC	7 (46.7)	5 (71.4)	0.3808
Atrio-pulmonary connection, *n* (%)	5 (33.3)	2 (28.6)	1.000
Pleural effusion after TCPC (>14 days), *n* (%)	11 (73.3)	7 (100)	0.2632
Hospital stay after TCPC (>14 days), *n* (%)	12 (80)	7 (100)	0.5227
History of clinically significant arrhythmia, *n* (%)	1 (6.7)	2 (28.6)	0.2273
History of clinically signs/symptoms of HF necessitating diuretic therapy, *n* (%)	5 (33.3)	6 (85.7)	0.0635
History of thromboembolic event, *n* (%)	1 (6.7)	4 (57.1)	0.0207 *

Data were described using mean (SD); SD: standard deviation or median (IQR: 25th percentile to 75th percentile) or absolute (relative frequencies,%); *p*-values were obtained from Student’s *t*-test for independent samples or Wilcoxon rank sum test with continuity correction or Fisher’s exact test; * statistical significance: *p* < 0.05. P-LVEF: preserved left ventricular ejection fraction; R-LVEF: reduced left ventricular ejection fraction; BMI: body mass index; BSA: body surface area; SBP: systolic blood pressure; DBP: diastolic blood pressure; DILV: double inlet left ventricle; TA: tricuspid atresia; PS: pulmonary stenosis; VSD: ventricular septal defect; AVC: atrioventricular canal; LPA: left pulmonary artery; RPA: right pulmonary artery; CoAo: coarctation of the aorta; AV: atrio-ventricular valve; PCPC: partial cavo-pulmonary connection; RA: right atrium, PAP: pulmonary arterial pressure; TCPC: total cavo-pulmonary connection; HF: heart failure; *n*: number.

**Table 2:** Hemodynamic, clinical, and paraclinical characteristics of the studied groups.

Variable	Fontan Patients with P-LVEF (*n* = 15)	Fontan Patients with R-LVEF (*n* = 7)	*p*-Value
Hemodynamic parameters			
PVRi (WU/m^2^), mean (SD)	1.69 (0.73)	2.63 (1.22)	0.0348 *
mPAP (mmHg), mean (SD)	10.20 (3.38)	13.14 (2.34)	0.0510
Nakata index (mm/m^2^), mean (SD)	294.10 (82.06)	236.40 (71.54)	0.1265
McGoon index, median (IQR)	2.17 (2.06 to 2.22)	1.85 (1.80 to 1.98)	0.0857
Frequent Clinical symptoms			
Cyanosis, *n* (%)	7 (46.7)	4 (57.1)	1.000
Edema, *n* (%)	0 (0)	3 (42.9)	0.0227 *
Hepatomegaly, *n* (%)	2 (13.3)	5 (71.4)	0.0136 *
EKG results, *n* (%)			
Sinus rhythm	13 (56.7)	4 (57.1)	0.2743
Atrial rhythm/junctional rhythm	2 (13.3)	2 (28.6)	0.5646
Paraclinical parameters			
WBC (cells/μL), median (IQR)	5890 (5650 to 8005)	6590 (5675 to 7980)	0.9437
RBC (cells/μL), median (IQR)	5.08 (4.96 to 5.29)	5.63 (5.14 to 5.93)	0.2171
Hgb (g/dL), mean (SD)	14.19 (0.99)	15.09 (1.16)	0.0769
HCT (%), mean (SD)	43.33 (2.94)	46.43 (2.98)	0.0331 *
Albumin (g/dL), mean (SD)	4.62 (0.44)	4.19 (0.90)	0.2614
gGT (U/L), median (IQR)	47 (35.5 to 62)	37 (31.5 to 41)	0.2588
ALT (U/L), mean (SD)	26.47 (8.62)	32.14 (9.53)	0.1789
AST (U/L), mean (SD	35.73 (8.57)	33 (9.64)	0.5103
NT-proBNP (pg/mL), median (IQR)	46.1 (37.8 to 116.5)	124.9 (38.8 to 219.3)	0.4475
Fe (μg/dL), median (IQR)	54 (50.5 to 76)	69 (49.5 to 77.5)	0.8320

Data were described using mean (SD); SD: standard deviation or median (IQR: 25th percentile to 75th percentile) or absolute (relative frequencies,%); *p*-values were obtained from Student’s *t*-test for independent samples or Wilcoxon rank sum test with continuity correction or Fisher’s exact test; * statistical significance: *p* < 0.05. P-LVEF: preserved left ventricular ejection fraction; R-LVEF: reduced left ventricular ejection fraction; PVRi: pulmonary vascular resistance index (Wood Units/meter square); mPAP: mean pulmonary artery pressure; EKG: electrocardiogram; WBC: white blood cell count; RBC: red blood cell count; Hgb; hemoglobin; HCT: hematocrit; gGT (U/L): gamma-glutamyl transferase; ALT (U/L): alanine aminotransferase; AST (U/L): aspartate aminotransferase; NT-proBNP: N-Terminal Pro-B-Type Natriuretic Peptide; Fe: serum iron level.

**Table 3:** Distributions of conventional left ventricle 2D and 3D echocardiographic parameters in the Fontan patients with R-LVEF and Fontan patients with P-LVEF.

Variable	Fontan Patients with P-LVEF (*n* = 15)	Fontan Patients with R-LVEF (*n* = 7)	*p*-Value
Mi_annulus (mm)	24.55 (3.40)	23.56 (5.68)	0.6137
MAPSE (mm)	12.26 (3.24)	12.63 (1.65)	0.7808
E_mi (m/s)	0.78 (0.66 to 0.87)	0.88 (0.69 to 1.00)	0.3065
A_mi (m/s)	0.64 (0.21)	0.76 (0.15)	0.2220
E_mi/A_mi	1.21 (0.54)	1.27 (0.39)	0.7766
E′_mi (cm/s)	15.79 (4.72)	13.17 (4.17)	0.2248
A′_mi (cm/s)	6.85 (6.27 to 10.19)	7.07 (6.58 to 9.43)	1.000
S_mi (cm/s)	8.10 (2.61)	6.58 (1.01)	0.0634
E_mi/E′_mi	0.04 (0.03 to 0.05)	0.05 (0.04 to 0.055)	0.6150
IVCT_mi (ms)	69 (66 to 76.5)	74 (65 to 78)	0.9150
IVRT_mi (ms)	82 (74 to 82)	71 (69 to 92)	0.8049
EDV (mL)	88 (78 to 116.5)	78 (69.5 to 109)	0.8050
ESV (mL)	35 (28.5 to 45.5)	51 (44.5 to 60)	0.0315 *
EDL (mm)	71.40 (9.90)	73.29 (15.79)	0.7344
ESL (mm)	57 (53 to 64)	69 (67.5 to 72)	0.0024 *
SV (mL)	54 (35 to 66.5)	31 (28 to 49.5)	0.2041
ED mass (g)	71 (56.5 to 94)	97 (63 to 118)	0.2743

Data are expressed as arithmetic mean (SD); SD: standard deviation or median (IQR:p 25th percentile to 75th percentile); Student’s *t*-test for independent samples or Wilcoxon rank sum test with continuity correction was performed to compare the groups; * statistical significance: *p* < 0.05. P-LVEF: preserved left ventricular ejection fraction; R-LVEF: reduced left ventricular ejection fraction; Mi: mitral; MAPSE (mm): mitral annular plain systolic excursion; E_mi (m/s): peak mitral inflow velocity during early diastole (E wave); A_mi (m/s): peak mitral inflow velocity at atrial contraction (A wave); E_mi/A_mi: the ratio between early mitral inflow velocity and late mitral inflow velocity; E′_mi (cm/s): mitral annular early diastolic velocity (E′wave); A′_mi (cm/s): mitral annular late diastolic velocity (A′wave); S_mi (cm/s): mitral annular systolic velocity (S wave); E_mi/E′_mi: the ratio between early mitral inflow velocity and mitral annular early diastolic velocity (E/E′); IVCT_mi (ms): left ventricle isovolumic contraction time; IVRT_mi (ms): left ventricle isovolumic relaxation time; EDV (mL): left ventricle end-diastolic volume: ESV (mL): left ventricle end-systolic volume; EDL (mm): left ventricle end-diastolic longitudinal diameter; ESL (mm): left ventricle end-systolic longitudinal diameter; SV (mL): left ventricle stroke volume, ED mass (g): left ventricle end-diastolic mass.

**Table 4:** Distributions of echocardiographic global longitudinal strain and segmental longitudinal strain indices across study groups.

Variable	Fontan Patients with P-LVEF (*n* = 15)	Fontan Patients with R-LVEF (*n* = 7)	*p*-Value
LV_BIS (%)	−14.74 (8.71)	−12.83 (7.46)	0.6225
LV_MIS (%)	−11.02 (5.21)	−11.70 (8.02)	0.8127
LV_AIS (%)	−11.57 (6.98)	−11.81 (5.14)	0.9361
LV_BAL (%)	−18.79 (10.81)	−14.21 (5.90)	0.3105
LV_MAL (%)	−15.95 (5.84)	−16.43 (3.21)	0.8435
LV_AAL (%)	−15.21 (6.32)	−17.04 (4.34)	0.4969
LV_BI (%)	−18.97 (13.63)	−21.16 (9.55)	0.7069
LV_MI (%)	−13.33 (5.76)	−14.17 (4.28)	0.7341
LV_AI (%)	−17.03 (9.15)	−13.73 (4.72)	0.3830
LV_BA (%)	−14.65 (7.42	−12.54 (11.44)	0.6071
LV_MA (%)	−10.19 (3.71)	−12.20 (9.23)	0.5967
LV_AA (%)	−15.26 (8.69)	−13.57 (7.25)	0.6610
LV_BIL (%)	−22.33 (9.71)	−16.94 (7.95)	0.2167
LV_MIL (%)	−12.76 (6.50)	−13.29 (10.47)	0.8859
LV_AL (%)	−13.11 (7.35)	−11.49 (8.13)	0.6445
LV_BAS (%)	−9.63 (6.64)	−7.69 (5.80)	0.5150
LV_MAS (%)	−7.41 (3.78)	−10.09 (7.36)	0.3916
LV_AA^2^ (%)	−9.86 (6.31)	−10.56 (6.55)	0.8139
LV_GLS_A4C (%)	−14.68 (2.05)	−13.94 (3.29)	0.5245
LV_GLS_A2C (%)	−14.56 (3.49)	−13.80 (3.83)	0.6491
LV_GLS_A3C (%)	−12.72 (3.25)	−11.97 (4.14)	0.6490
LV_GLS (%)	−14.18 (2.08)	−13.00 (3.22)	0.3120
LA_Sr_ED (%)	23.95 (9.17)	30.43 (8.12)	0.1265
LA_Scd_ED (%)	−15.43 (8.48)	−20.64 (8.81)	0.1998
LA_Sct_ED (%)	−11.02 (9.74)	−9.37 (6.18)	0.6875
LA_Sr_AC (%)	22.02 (8.49)	28.51(6.99)	0.0941
LA_Scd_AC (%)	−14.33 (8.26)	−19.43 (8.32)	0.1936
LA_Sct_AC (%)	−7.80 (3.85)	−9.09 (5.91)	0.5455

P-LVEF: preserved left ventricular ejection fraction; R-LVEF: reduced left ventricular ejection fraction; LV: left ventricle; BIS: basal inferoseptal; MIS: mid inferoseptal; AIS: apical inferoseptal; BAL: basal anterolateral; MAL: mid anterolateral; AAL: apical anterolateral; BI: basal inferior; MI: mid inferior; AI: apical inferior; BA: basal anterior; MA: mid anterior; AA: apical anterior (assessed from apical two chamber view); BIL: basal inferolateral; MIL: mid inferolateral; AL: apical lateral; BAS: basal anteroseptal; MAS: mid anteroseptal; AA^2^: apical anterior (assessed from apical three chamber view); LV_GLS_A4C: left ventricular apical four chamber longitudinal strain; LV_GLS_A2C: left ventricular apical two chamber longitudinal strain; LV_GLS_A3C: left ventricular apical three chamber longitudinal strain; LV_GLS: left ventricular global longitudinal strain; LA_Sr_ED: left atrial reservoir strain by using the starting points of R-wave peak; LA_Scd_ED: left atrial conduit strain by using the starting points of R-wave peak; LA_Sct_ED: left atrial contractile strain by using the starting points of R-wave peak; LA_Sr_AC: left atrial reservoir strain by using the starting points of P-wave onset; LA_Scd_AC: left atrial conduit strain by using the starting points of P-wave onset; LA_Sct_AC: left atrial contractile strain by using the starting points of P-wave onset. We used Student’s *t*-test for independent samples; statistical significance: *p* < 0.05.

**Table 5:** Echocardiographic global longitudinal strain and segmental longitudinal strain indices by cardiac morphology.

Variable	Fontan Patients with DILV (*n* = 7)	Fontan Patients with TA+PS+VSD (*n* = 9)	Fontan Patients with Unbalanced AVC (*n* = 6)	*p*-Value
LV_BIS (%)	−14.50 (10.85)	−15.67 (6.72)	−11.40 (7.46)	0.6310
LV_MIS (%)	−8.64 (5.80)	−14.63 (6.02)	−9.17 (4.46)	0.0834
LV_AIS (%)	−6.83 (4.45)	−13.81 (7.00)	−14.03 (4.26)	0.0419 *
LV_BAL (%)	−21.11 (8.61)	−12.40 (5.55)	−20.32 (13.27)	0.1320
LV_MAL (%)	−16.99 (5.48)	−14.60 (5.07)	−17.33 (4.88)	0.5300
LV_AAL (%)	−15.41 (6.60)	−16.53 (4.62)	−15.12 (7.03)	0.8860
LV_BI (%)	−19.71 (7.82)	−21.19 (9.39)	−24.05 (8.65)	0.6700
LV_MI (%)	−11.71 (4.02)	−16.70 (4.77)	−11.13 (5.50)	0.0602
LV_AI (%)	−15.76 (11.95)	−17.32 (6.01)	−14.22 (5.93)	0.7790
LV_BA (%)	−14.91 (7.21)	−18.69 (8.52)	−5.83 (4.04)	0.0102 *
LV_MA (%)	−13.77 (7.18)	−10.30 (5.66)	−8.20 (3.27)	0.2270
LV_AA (%)	−17.79 (10.39)	−12.23 (6.59)	−14.88 (7.36)	0.4180
LV_BIL (%)	−19.73 (7.20)	−17.99 (8.63)	−25.58 (11.97)	0.3060
LV_MIL (%)	−10.33 (7.41)	−16.61 (7.75)	−10.43 (6.81)	0.1790
LV_AL (%)	−9.40 (6.43)	−13.29 (7.53)	−15.28 (8.29)	0.3590
LV_BAS (%)	−11.39 (5.86)	−9.08 (7.90)	−6.13 (2.91)	0.3430
LV_MAS (%)	−8.33 (7.71)	−7.89 (4.42)	−8.73 (2.93)	0.9560
LV_AA^2^ (%)	−8.53 (6.66)	−9.50 (6.23)	−12.77 (5.96)	0.4660
LV_GLS_A4C (%)	−14.79 (2.63)	−14.68 (1.75)	−13.70 (3.33)	0.7010
LV_GLS_A2C (%)	−14.17 (5.10)	−15.52 (1.83)	−12.68 (3.14)	0.3240
LV_GLS_A3C (%)	−11.46 (3.81)	−12.63 (3.75)	−13.45 (2.85)	0.6020
LV_GLS (%)	−13.88 (3.07)	−14.28 (1.58)	−13.00 (3.07)	0.6400
LA_Sr_ED (%)	26.83 (12.24)	24.61 (8.34)	27.17 (7.60)	0.8490
LA_Scd_ED (%)	−19.91 (13.60)	−14.77 (4.93)	−17.28 (6.24)	0.5270
LA_Sct_ED (%)	−12.29 (13.89)	−9.96 (5.83)	−9.42 (4.09)	0.8200
LA_Sr_AC (%)	25.26 (11.95)	22.16 (6.86)	25.62 (6.55)	0.6920
LA_Scd_AC (%)	−18.93 (13.29)	−13.46 (4.54)	−16.23 (5.47)	0.4560
LA_Sct_AC (%)	−6.40 (3.83)	−8.83 (4.83)	−9.38 (4.81)	0.4450

DILV: double inlet left ventricle; TA: tricuspid atresia; PS: pulmonary stenosis; VSD: ventricular septal defect; AVC: atrioventricular canal; LV: left ventricle; BIS: basal inferoseptal; MIS: mid inferoseptal; AIS: apical inferoseptal; BAL: basal anterolateral; MAL: mid anterolateral; AAL: apical anterolateral; BI: basal inferior; MI: mid inferior; AI: apical inferior; BA: basal anterior; MA: mid anterior; AA: apical anterior (assessed from apical two chamber view); BIL: basal inferolateral; MIL: mid inferolateral; AL: apical lateral; BAS: basal anteroseptal; MAS: mid anteroseptal; AA^2^: apical anterior (assessed from apical three chamber view); LV_GLS_A4C: left ventricular apical four chamber longitudinal strain; LV_GLS_A2C: left ventricular apical two chamber longitudinal strain; LV_GLS_A3C: left ventricular apical three chamber longitudinal strain; LV_GLS: left ventricular global longitudinal strain; LA_Sr_ED: left atrial reservoir strain by using the starting points of R-wave peak; LA_Scd_ED: left atrial conduit strain by using the starting points of R-wave peak; LA_Sct_ED: left atrial contractile strain by using the starting points of R-wave peak; LA_Sr_AC: left atrial reservoir strain by using the starting points of P-wave onset; LA_Scd_AC: left atrial conduit strain by using the starting points of P-wave onset; LA_Sct_AC: left atrial contractile strain by using the starting points of P-wave onset. *p*-values were obtained from one-way ANOVA analysis or Welch F-test; * statistical significance: *p* < 0.05.

**Table 6:** Two-dimensional and three-dimensional echocardiographic characteristics according to congenital malformations type.

Variable	Fontan Patients with DILV (*n* = 7)	Fontan Patients with TA+PS+VSD (*n* = 9)	Fontan Patients with Unbalanced AVC (*n* = 6)	*p*-Value
Mi_annulus (mm)	24.21 (3.94)	26.38 (3.25)	21.03 (3.98)	0.0410 *
MAPSE (mm)	13.73 (2.83)	11.73 (3.25)	11.77 (1.56)	0.3150
E_mi (m/s)	0.94 (0.16)	0.69 (0.13)	0.93 (0.55)	0.0220 *
A_mi (m/s)	0.75 (0.25)	0.58 (0.14)	0.75 (0.14)	0.1410
E_mi/A_mi	1.35 (0.38)	1.26 (0.41)	1.04 0.69)	0.5250
E′_mi (cm/s)	15.26 (4.11)	15.99 (5.15)	13.05 (4.54)	0.4950
A′_mi (cm/s)	6.34 (6.21 to 8.21)	8.81 (6.96 to 11.30)	6.83 (6.38 to 7.81)	0.3978
S_mi (cm/s)	7.55 (2.89)	8.61 (2.21)	6.22 (0.74)	0.0353 *
E_mi/E′_mi	0.05 (0.04 to 0.07)	0.04 (0.03 to 0.05)	0.045 (0.04 to 0.06)	0.5320
IVCT_mi (ms)	66 (63.5 to 84.5)	69 (66 to 74)	80.5 (71.5 to 82)	0.4179
IVRT_mi (ms)	90 (79.5 to 100)	71 (63 to 77)	70 (66.8 to 79.3)	0.1397
EDV (mL)	115 (94 to 135)	87 (74 to 119)	74.5 (66.5 to 81)	0.0760
ESV (mL)	42 (34.5 to 67)	35 (29 to 56)	44.5 (36.8 to 50.8)	0.6838
EDL (mm)	79.29 (13.48)	67 (10.17)	71 (8.53)	0.1080
ESL (mm)	66.29 (13.51)	58.33 (6.98)	65.83 (6.55)	0.1950
SV (mL)	62.43 (31.94)	53 (15.97)	31.67 (8.50)	0.0099 *
ED mass (g)	82 (49.5 to 94)	99 (69 to 129)	67 (56 to 84.75)	0.4767

Data are expressed as arithmetic mean (SD); SD: standard deviation or median (IQR: 25th percentile to 75th percentile); *p*-values were obtained from one-way ANOVA analysis or Welch F-test or Kruskal–Wallis test; * statistical significance: *p* < 0.05. DILV: double inlet left ventricle; TA: tricuspid atresia; PS: pulmonary stenosis; VSD: ventricular septal defect; AVC: atrioventricular canal; Mi_annulus: mitral annulus; MAPSE (mm): mitral annular plain systolic excursion; E_mi (m/s): peak mitral inflow velocity during early diastole (E wave); A_mi (m/s): peak mitral inflow velocity at atrial contraction (A wave); E_mi/A_mi: the ratio between early mitral inflow velocity and late mitral inflow velocity; E′_mi (cm/s): mitral annular early diastolic velocity (E′wave); A′_mi (cm/s): mitral annular late diastolic velocity (A′wave); S_mi (cm/s): mitral annular systolic velocity (S wave); E_mi/E′_mi: the ratio between early mitral inflow velocity and mitral annular early diastolic velocity (E/E′); IVCT_mi (ms): left ventricle isovolumic contraction time; IVRT_mi (ms): left ventricle isovolumic relaxation time; EDV (mL): left ventricle end-diastolic volume: ESV (mL): left ventricle end-systolic volume; EDL (mm): left ventricle end-diastolic longitudinal diameter; ESL (mm): left ventricle end-systolic longitudinal diameter; SV (mL): left ventricle stroke volume, ED mass (g): left ventricle end-diastolic mass.

## Data Availability

The original contributions presented in this study are included in the article. Further inquiries can be directed to the corresponding author.

## Abbreviations

The following abbreviations are used in this manuscript:

AA	Apical Anterior
AAL	Apical Anterolateral
AA2	Apical Anterior (2-/3-chamber view)
A_mi	Peak Mitral Inflow Velocity at Atrial Contraction (A wave)
A′_mi	Mitral Annular Late Diastolic Velocity
AC	Atrial Contraction (P-wave reference)
AIS	Apical Inferoseptal
AL	Apical Lateral
ALT	Alanine Aminotransferase
AST	Aspartate Aminotransferase
AV	Atrioventricular Valve
AVC	Atrioventricular Canal
BA	Basal Anterior
BAL	Basal Anterolateral
BAS	Basal Anteroseptal
BI	Basal Inferior
BIL	Basal Inferolateral
BIS	Basal Inferoseptal
BMI	Body Mass Index
BSA	Body Surface Area
CMR	Cardiac Magnetic Resonance
CoAo	Coarctation of the Aorta
DBP	Diastolic Blood Pressure
DILV	Double Inlet Left Ventricle
ED	End-Diastole
EDL	End-Diastolic Longitudinal Diameter
EDV	End-Diastolic Volume
ED mass	End-Diastolic Mass
EF	Ejection Fraction
EKG	Electrocardiogram
ESL	End-Systolic Longitudinal Diameter
ESV	End-Systolic Volume
E_mi	Peak Mitral Inflow Velocity During Early Diastole
E_mi/A_mi	Ratio of Early to Late Mitral Inflow Velocities
E′_mi	Mitral Annular Early Diastolic Velocity
E_mi/E′_mi	Ratio of E to E′
FC	Functional Class (NYHA)
Fe	Serum Iron
GLS	Global Longitudinal Strain
gGT	Gamma-Glutamyl Transferase
HCT	Hematocrit
Hgb	Hemoglobin
HF	Heart Failure
IVCT	Isovolumic Contraction Time
IVRT	Isovolumic Relaxation Time
LA	Left Atrium/Left Atrial
LA_Sr	Left Atrial Reservoir Strain
LA_Scd	Left Atrial Conduit Strain
LA_Sct	Left Atrial Contractile Strain
LV	Left Ventricle/Left Ventricular
LV_AI	LV Apical Inferior
LV_AA	LV Apical Anterior
LV_AAL	LV Apical Anterolateral
LV_AIS	LV Apical Inferoseptal
LV_AL	LV Apical Lateral
LV_BA	LV Basal Anterior
LV_BAL	LV Basal Anterolateral
LV_BAS	LV Basal Anteroseptal
LV_BI	LV Basal Inferior
LV_BIL	LV Basal Inferolateral
LV_BIS	LV Basal Inferoseptal
LV_MA	LV Mid Anterior
LV_MAL	LV Mid Anterolateral
LV_MAS	LV Mid Anteroseptal
LV_MI	LV Mid Inferior
LV_MIL	LV Mid Inferolateral
LV_MIS	LV Mid Inferoseptal
LV_GLS	LV Global Longitudinal Strain
LV_GLS_A2C	LV GLS from Apical Two-Chamber
LV_GLS_A3C	LV GLS from Apical Three-Chamber
LV_GLS_A4C	LV GLS from Apical Four-Chamber
mPAP	Mean Pulmonary Artery Pressure
MAPSE	Mitral Annular Plane Systolic Excursion
Mi_annulus	Mitral Annulus Diameter
NT-proBNP	N-Terminal pro–B-Type Natriuretic Peptide
NYHA	New York Heart Association
P-LVEF	Preserved Left Ventricular Ejection Fraction
PCPC	Partial Cavo-Pulmonary Connection
PVRi	Pulmonary Vascular Resistance Index
PS	Pulmonary Stenosis
R-LVEF	Reduced Left Ventricular Ejection Fraction
RA	Right Atrium
RBC	Red Blood Cell Count
RPA	Right Pulmonary Artery
RV	Right Ventricle/Right Ventricular
SBP	Systolic Blood Pressure
SD	Standard Deviation
S_mi	Mitral Annular Systolic Velocity
STE	Speckle-Tracking Echocardiography
SV	Stroke Volume/Single Ventricle
TA	Tricuspid Atresia
TCPC	Total Cavo-Pulmonary Connection
VSD	Ventricular Septal Defect
WBC	White Blood Cell Count
WU	Wood Units
